# Decomposition analysis of LTREs may facilitate the design of short-term ecotoxicological tests

**DOI:** 10.1007/s10646-012-0904-5

**Published:** 2012-04-21

**Authors:** Natnael T. Hamda, Dragan M. Jevtić, Ryszard Laskowski

**Affiliations:** Institute of Environmental Sciences, Jagiellonian University, Gronostajowa 7, 30-387 Kraków, Poland

**Keywords:** Life table response experiment, Matrix population model, Decomposition analysis, Elasticity, *Acyrthosiphon pisum*, Ecotoxicological assays

## Abstract

This study compared two methods, based on re-analyzed data from a partly published life table response experiment (LTRE), to help determine the optimal approach for designing ecotoxicological assessments. The 36-day LTRE data recorded the toxic effects of cadmium (Cd) and imidacloprid, alone and in combination, on the reproduction and survivorship of aphids (*Acyrthosiphon pisum* Harris). We used this data to construct an age-classified matrix model (six age classes, each 6 days long) to estimate aphid population growth rate (λ) under each treatment. For each treatment, an elasticity analysis and a demographic decomposition analysis were performed, and results were compared. Despite different results expected from the two toxicants, the elasticity values were very similar. The elasticity of λ with respect to survival was highest in the first age class, and that with respect to fertility was highest in the second age class. The demographic decomposition analysis examined how changes in life-history traits contributed to differences in λ between control and treated populations (Δλ). This indicated that the most important contributors to Δλ were the differences in survival (resulting from both demographic sensitivity and toxicity) in the first and the second age classes of aphids and differences in fertility in the third and the fourth age classes. Additionally, the toxicants acted differently. Cd reduced Δλ by impairing fertility at third age class and reducing survivorship from the second to the third age class. Imidacloprid mostly reduced survivorship at the first and second age classes. The elasticity and decomposition analyses showed different results, because these methods addressed different questions about the interaction of organism life history and sensitivity to toxicants. This study indicated that the LTRE may be useful for designing individual-level ecotoxicological experiments that account for both the effects of the toxicant and the demographic sensitivity of the organism.

## Introduction

The ecological risks of toxic chemicals are typically assessed on the basis of individual survival, reproduction, or growth. These data might be more effectively used to predict the effects of toxic chemicals at higher levels of ecological organization. In fact, the protection aims of various Ecological Risk Assessment (ERA) schemes commonly focus on populations, communities, or entire ecosystems (Hommen et al. 2010). Thus, the impact of toxicants on an individual life history is typically extended to the population level with mathematical models designed to calculate the main demographic indices and other population statistics. In particular, the population growth rate (λ), which takes into account survival, development time, and reproduction, can provide valuable information that cannot be obtained from the analysis of single parameters of individual life history. Previously, Forbes and Calow (1999, 2002) and Barnthouse et al. (2008) discussed the suitability of using population growth rate as a measure of population-level toxicant effects. Among different mathematical modeling approaches, one of the most robust techniques is matrix population modeling. In that approach, individual level data is used as an input to calculate characteristic endpoints for the population (Caswell 1997, 2001).

Traditional ecotoxicological testing often determines a dose–effect relationship for a given chemical on a certain endpoint (like survival or reproduction) at a fixed time point. Although these tests represent a standard in ecotoxicology, the data generated are only applicable to effects on the measured endpoint at one specific time point. These short-term assays have several drawbacks for environmental risk assessments, which have been well scrutinized by various authors. For example, Laskowski (2001) showed that these short-term assays neglected the cumulative nature of some chemicals and the potential accumulation of toxic effects. Laskowski (2001) also pointed out that short-term assays neglect effects on outcomes other than mortality and fecundity and account for only a small part of the life history of an organism. Therefore, they do not provide any information on population dynamics under toxic stress. On the other hand, conducting a full life cycle study in a life table response experiment (LTRE) is lengthy, impractical, and expensive. Thus, there is a need for ecotoxicological tests that can more efficiently capture toxic chemical effects on the population level, based on individual-level responses. Demographic perturbation analysis can be a powerful tool for developing such tests. It explores how population statistics respond to changes in the vital rates using two logically distinct ways (Caswell 2000). Prospective analyses (e.g. elasticity) explore the functional relationship between population growth rate and the vital rates, while retrospective analyses (e.g. demographic decomposition of LTREs) enables the variation in λ to be expressed as a function of variation in vital rates (Caswell 1996a, 2000).

The extent to which λ responds to toxicant exposure depends on the severity of the toxicant effect on individual life-history traits and the sensitivity of λ to changes in the individual traits that contribute to it (Levin et al. 1996). Hence, based on the information obtained from a demographic decomposition of LTRE, we can pinpoint the most ecotoxicologically important life stages of an organism; i.e. the life stages that capture most of the severity of toxicant’s effect on λ. This technique can be considered an empirical extension of a sensitivity analysis; it allows one to analyze data from chronic toxicity tests to assess how the toxicant impact on each vital rate contributes to the overall impact on population growth (Laskowski 1997).

Due to the broad use of some of the terms used in this area, we would like to define the following terms: *elasticity* in this work refers to the (mathematical) demographic sensitivity of λ to changes in vital rates; and *contribution* represents the combined effects of demographic sensitivity and the response of vital rates to toxicants on λ. These strict definitions of terms are intended to avoid subsequent confusion.

The main objective of this study was to compare results from retrospective (demographic decomposition of LTRE) and prospective (elasticity analysis) approaches to highlight potential differences in outputs and the potential consequences of applying each method in ecotoxicology testing and risk assessment. The demographic decomposition of LTRE data also allowed a comparison of the contributions of each vital rate to the actual changes in the population growth rate in treated and control groups (Δλ). The results were used to identify the most important aphid life stages that, when investigated at the individual level, can capture population-level impacts of toxicants. Because the data originated from the full factorial experiment, we were able to perform a comparison between single-chemical treatments and combined-chemical treatments.

## Materials and methods

### Experimental design

The data for this study originate from an experiment performed by Laskowski (2001). Briefly, the aphids (*Acyrthosiphon pisum* Harris) were bred on potted broad bean plants, *Vicia faba* L., at 18 °C, under a light:dark regime of 16:8 h. In each pot, four plants were planted in 250 g dry weight garden soil. The full factorial design was used, with two Cd treatments (100 and 200 mg kg^−1^ dry weight), two imidacloprid treatments (4 and 40 g a.i. ha^−1^) and the four possible combined treatments assigned among the pots at random before planting the beans. Cd contamination was achieved by watering the soil with 100 ml of an appropriate CdCl_2_ solution (anhydrous, ACS grade in distilled water) before the beans were planted. Controls were treated with an equal volume of distilled water. After 3 weeks, imidacloprid contamination was achieved by adding imidacloprid (BAY NTN 33893, 17.4 % active ingredient) in 50 ml distilled water directly to the soil surface of each pot. The doses were approximately equivalent to 0.01× and 0.1× of the recommended field dose. This resulted in nominal concentrations of ca. 14.4 and 144 ng a.i. g^−1^ of dry soil, respectively. Controls and other treatments received 50 ml distilled water. Four days later, only three plants per pot were kept, and aphids were introduced to the plants. The adult reproducing aphids were placed individually into clip cages, one per pot; after 24 h, the adults and all neonates except one per cage were removed. Every clip cage was monitored daily until the death of the aphid; and all newborn neonates were counted and removed. When the leaf with a clip cage started to wilt, the cage and aphid were moved to a new leaf; when the whole plant wilted, they were moved to another plant in the same pot. Throughout the experiment, the pots with plants remained in plastic trays filled with tap water. The experiment ended after 36 days, when all the aphids died.

### The matrix model

An age-structured matrix projection model, known as the Leslie matrix model, was used to investigate the population-level effects of Cd and imidacloprid applied alone and in mixtures. For this, the individual daily life-history data of aphids were recalculated and organized in cohort life tables. The aphids lived for a maximum of 36 days; hence, we used life tables with 6 age classes, each 6 days long (i.e., 1–6, 7–12,…,31–36 days). The life tables were used to parameterize age-classified projection matrices, and the entries were rounded to the nearest second decimal digit for survival rates.

The basic structure of the Leslie matrix is given in Eq. 1.

1 $$ \left[ {\begin{array}{*{20}c} {F_{1} } & {F_{2} } & {F_{3} } & {F_{4} } & {F_{5} } & {F_{6} } \\ {P_{1} } & 0 & 0 & 0 & 0 & 0 \\ 0 & {P_{2} } & 0 & 0 & 0 & 0 \\ 0 & 0 & {P_{3} } & 0 & 0 & 0 \\ 0 & 0 & 0 & {P_{4} } & 0 & 0 \\ 0 & 0 & 0 & 0 & {P_{5} } & 0 \\ \end{array} } \right]*\left[ \begin{gathered} N_{1} (t) \hfill \\ N_{2} (t) \hfill \\ N_{3} (t) \hfill \\ N_{4} (t) \hfill \\ N_{5} (t) \hfill \\ N_{6} (t) \hfill \\ \end{gathered} \right] = \left[ \begin{aligned} N_{1} (t + 1) \hfill \\ N_{2} (t + 1) \hfill \\ N_{3} (t + 1) \hfill \\ N_{4} (t + 1) \hfill \\ N_{5} (t + 1) \hfill \\ N_{6} (t + 1) \hfill \\ \end{aligned} \right] $$

*F*_i_ represents the fecundity of age classes 1–6; *P*_i_ represents the probability of survival from age class *i* to *i* + 1; and *N*_*i*_(t) and *N*_*i*_(*t* + 1) represent the numbers of individuals in age class *i* at time *t* and *t* + 1, respectively.

### Elasticity analysis

Elasticity analysis explores the functional dependence of λ on the individual vital rates. It has traditionally been used to determine to what extent particular vital rates are important for population dynamics (measured by λ) of a species characterized by a specific life history. Thus, the elasticity of a matrix element, *ε*_*ij*_, is the product of the sensitivity of a matrix element ($S_{ij}$) times the matrix element itself ($a_{ij}$), divided by λ. In essence, elasticities are proportional sensitivities, dimensionless, and normalized to between 0 and 1 to facilitate comparison among vital rates with different scales.

Mathematically, the sensitivity $\frac{\partial \lambda }{{\partial a_{ij} }}$ can be calculated as follows (Caswell 2001):

2 $$ \frac{\partial \lambda }{{\partial a_{ij} }} = \frac{{\bar{v}_{i} w_{j} }}{{\left\langle {w,v} \right\rangle }} $$

where ***w*** and ***v*** are corresponding right and left eigenvectors of the largest eigenvalue, λ. Accordingly, elasticity of a matrix element can be calculated as follows (Caswell 2001):

3a $$ \varepsilon_{ij} = \frac{\partial \log \lambda }{{\partial \log a_{ij} }} $$

or

3b $$ \varepsilon_{ij} = \frac{{a_{ij} }}{\lambda }\left( {\frac{\partial \lambda }{{\partial a_{ij} }}} \right) . $$

Equations 3a and 3b are used here to calculate the elasticities for an untreated population. In addition, elasticities were calculated for each treated population (expressed in percentage):

3c $$ \varepsilon_{ij}^{T} (\% ) = \frac{{a_{ij}^{T} }}{{\lambda^{T} }}\left( {\frac{{\partial \lambda^{T} }}{{\partial a_{ij}^{T} }}} \right)*100 $$

The superscript *T* represents each treatment level.

This elasticity analysis captured the toxic effects, and the output could be compared to results from the demographic decomposition of the LTRE.

### Demographic decomposition of LTRE

Demographic decomposition methods express observed variations in λ as a function of observed (co)variations in the vital rates. The differences in population growth rates (Δ*λ*) between the treated and control groups were analyzed according to the contributions of age-specific fecundities and survival probabilities. These contributions were identified based on the decomposition analysis technique proposed by Caswell (1996a), which was based on the Taylor’s series expansion technique. In this formulation, *λ* ^(*T*)^ and *λ* ^(*C*)^ denote the values of *λ* for treated and control groups, respectively. Then, the first order approximation of *λ* for toxicant-treated groups is given by taking *λ* ^(*C*)^ as the base of expansion, as follows:

4 $$ \Updelta \lambda = \lambda^{(T)} - \lambda^{(C)} \approx \sum\limits_{i,j} {\left( {a_{ij}^{(T)} - a_{ij}^{(C)} } \right)} \frac{\partial \lambda }{{\partial a_{ij} }}\left| {_{{\frac{1}{2}\left( {A^{(T)} + A^{(C)} } \right)}} } \right. $$

In the summation, the difference between treated and control values in each Leslie matrix element, *a*_ij_ (age-specific survivorship or fecundity values) was evaluated for its contribution to the global difference in the population growth rate, ∆λ. In this way, it was possible to identify the life-history traits that had the greatest influence on λ for each treatment and compare the relative contributions of each trait to the ∆λ between the treated and control groups.

As mentioned above and confirmed by Caswell (1996a) and Levin et al. (1996), Eq. 4 is an approximation; its accuracy should be verified prior to implementation to determine whether a higher order approximation should be used. Therefore, we conducted a preliminary analysis aimed at testing the accuracy of the equation to be applied to our LTRE by comparing the equivalency of the left and right hand sides (LHS & RHS) of Eq. 4.

The key to breaking-down the differences in population growth rates between the treated and control groups was to evaluate the terms inside the summation on the RHS of Eq. 4. The RHS of Eq. 4 contains two components. First, we calculated the difference between the matrix coefficients for the control and treated groups, $\left( {a_{ij}^{(T)} - a_{ij}^{(C)} } \right)$; then, we evaluated the sensitivity of each matrix coefficient with respect to the λ value at the midpoint between the control and treated groups, $\frac{\partial \lambda }{{\partial a_{ij} }}\left| {_{{\frac{1}{2}\left( {A^{(T)} + A^{(C)} } \right)}} } \right.$. The former is a straightforward subtraction. The latter calculation is equivalent to Eq. 2, where the sensitivity is calculated at the midpoint between the control and treated groups. Thus, we needed to evaluate the λ value at the midpoint between the control and each treatment; then, we could evaluate the corresponding right and left eigenvectors.

All calculations were performed with a program code developed in MATLAB. The built-in functions for matrix algebra were utilized to evaluate the demographic statistics (*λ*, *w*, and *v*), the sensitivities, and the contributions to treatment effects on λ.

## Results

### Parameter estimation

The individual life-history data were used to evaluate parameters in Eq. 1. Once the values of *F*_*i*_ and *P*_*i*_ were determined, we developed Leslie matrices for each treatment. In the individual life history data, large differences were found between individual lifetimes and fecundity. Consequently, there was also a large variation in calculated population growth rates. Therefore, a bootstrapping procedure was applied, where the data were reassembled 5,000 times. Table 1 summarizes the vital rates analysis results and the estimated population growth rates.

**Table 1:** Probability of survival, P_i_, fecundity, F_i_, and mean population growth rate, λ (day^-1^) for different treatments

Age class	Treatment levels^a^								
	0/0	100/0	200/0	0/4	0/40	100/4	200/4	100/40	200/40
*P*_*i*_									
1	0.89	0.90	0.68	0.50	0.46	0.74	0.88	0.33	0.71
2	0.94	0.83	0.54	0.83	0.67	0.86	0.71	0.75	0.80
3	0.77	0.53	0.29	0.60	0.75	0.42	0.30	0.33	0.38
4	0.54	0.25	0.00	0.33	0.33	0.00	0.00	0.00	0.00
5	0.31	0.00		0.00	0.00				
6	0.00								
*F*_*i*_									
1	1.5	2.3	0.8	3.7	0.0	2.4	2.3	0.0	0.4
2	50.2	44.4	29.6	39.7	12.8	41.9	35.0	11.5	19.6
3	27.0	19.8	8.5	11.7	18.5	16.9	8.6	11.0	15.5
4	1.6	1.9	0.0	0.5	0.0	0.3	0.0	0.0	0.7
5	0.0	0.0	0.0	0.0	0.0	0.0	0.0	0.0	0.0
6	0.0	0.0	0.0	0.0	0.0	0.0	0.0	0.0	0.0
Lambda, day^−1^ (values in brackets represent standard deviations)									
	1.282 (0.117)	1.274 (0.172)	0.829 (0.196)	1.125 (0.237)	0.469 (0.198)	1.172 (0.208)	1.148 (0.195)	0.373 (0.164)	0.701 (0.222)

^a^Treatment levels are represented as the Cd concentration in mg kg^−1^/imidacloprid dose in g a.i. ha^−1^

### Elasticity analysis

**Fig. 1:**
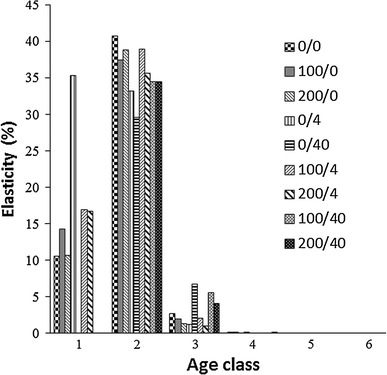
Control and treatment-specific lambda elasticities to changes in reproduction rates. The treatments are represented as Cd concentrations in mg kg^−1^ and imidacloprid dose in g a.i. ha^−1^; 0/0 stands for control

**Fig. 2:**
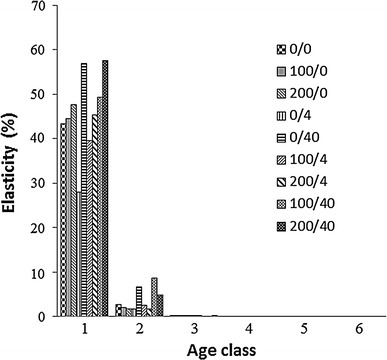
Control and treatment-specific lambda elasticities to changes in survival probabilities. The treatments are represented as Cd concentrations in mg kg^−1^ and imidacloprid dose in g a.i. ha^−1^; 0/0 stands for control

The elasticity analysis was first performed with a ‘traditional’ method; that is, the elasticities were calculated for an untreated population (Figs. 1, 2). The elasticity of λ with respect to survival was highest in the first age class, and that with respect to fertility was highest in the second age class. The elasticity with respect to fertility was also high in the first age class, despite the low reproduction rate compared to the second and third age classes (Table 1).

The fertility elasticities were also calculated for each treatment separately (Fig. 1). In the second age class, λ was highly sensitive to changes in fertility with all treatments. In the first age class, λ was sensitive to changes in fertility under all treatments, except the three with the high imidacloprid dose. This was a direct result of the fact that aphids did not reproduce at all in the first age class at imidacloprid = 40 g a.i. ha^−1^ (Table 1). For all other age classes, the λ sensitivity to changes in fertility was very low or zero with all treatments. A different pattern emerged for treatment-specific survival elasticities (Fig. 2). In the first age class, λ was highly sensitive to changes in survival with all treatments. For the second age class, the λ sensitivity was very low; for all other age classes, the effects were negligible.

### Accuracy analysis of numerical approximations

As mentioned above, prior to the detailed mathematical formulation of the LTRE analysis, we evaluated the accuracy of the first order numerical approximation shown in Eq. 4. The accuracy of the first order approximation was evaluated by comparing the left and right hand sides (LHS & RHS) of Eq. 4. The accuracy was reflected in the fit of the calculated values to the actual data. The results showed that the largest difference between the approximated values and the actual data was 0.189. Except for the treatment level of 200/40, the calculated values did not differ from the actual data by more than 3 %. This value is estimated to be 10.9 % for the 200/40 treatment level. Therefore we assumed the first order expansion to be good approximation of the differences in population growth rates between the treated and control groups.

Based on this first order approximation, we calculated the contribution of each matrix element to the global difference in population growth rate, Δλ, and the difference between each treatment and the control group was evaluated. Thus, it was possible to identify the life-history traits that had the greatest influence on λ, considering specific treatment effects. Then, we could compare the relative contributions of each vital rate to the difference in *λ* between the treated and control groups. Accordingly, the LTRE decomposition results provided the basis for understanding which life-history traits were most suitable for focused, short-term toxicity tests. The results pinpointed the stage-wise contributions of toxic effects on fertility and survival.

### Effect on fertility

All cadmium (Cd) treatments had a negative effect on aphid fertility. At the low Cd concentration (100 mg/kg), the effect on fertility was limited to the third and fourth age classes. At the higher concentration (200 mg/kg), the effects went beyond the third and fourth age classes, and reduced the fertility from the second to the fifth age classes. However, the effects in the second and fifth age classes were smaller than those in the third and fourth age classes.

Generally, imidacloprid also had a negative effect on aphid fertility. However, at the low treatment rate (4 ng a.i. ha^−1^), the effect was quite surprising. At this low level, imidacloprid affected the fertility of aphids positively in the second age class, and negatively in the third, fourth, and fifth age classes. The negative effect was strong in the third and fourth age classes. At the higher imidacloprid concentration (40 ng a.i. ha^−1^), the fertility of aphids was reduced further, and the effect was extended from the second to the fifth age class. The effect was most pronounced in the third and fourth age classes, particularly in the third age class.

In the combination of Cd and imidacloprid, the effect on fertility was most pronounced in the third and fourth age classes. The effect increased abruptly at the high concentration of imidacloprid.

**Fig. 3:**
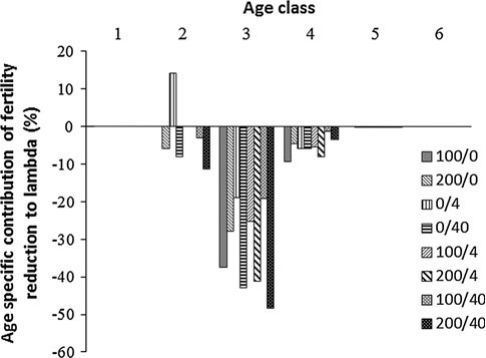
Treatment-specific contributions of reductions in fertility to the change in lambda. The treatments are represented as Cd concentrations in mg kg^−1^ and imidacloprid dose in g a.i. ha^−1^

The analysis of the proportional contributions of these effects to the population growth rate (λ) clearly indicated that both contaminants had the largest effects on the reproduction rate in the third age class, which significantly contributed to λ (Fig. 3). This was due to a combination of the strong effects of the contaminants on aphid fertility at this age and the heightened sensitivity of the population growth rate (λ) to changes in aphid fertility in the third age class (cf. elasticity analysis above).

### Effect on survival probability

**Fig. 4:**
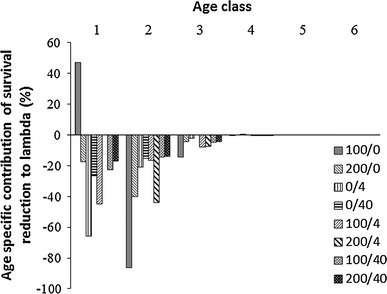
Treatment-specific contributions of survival reduction to the change in lambda. The treatments are represented as Cd concentrations in mg kg^−1^ and imidacloprid dose in g a.i. ha^−1^

In general, both Cd and imidacloprid had negative impacts on the survival of aphids. At the low Cd concentration (100 mg/kg), mortality decreased in the first age class compared to controls. Unlike the effects on fertility, the negative effects of these contaminants on survival were pronounced for all age classes. However, because the age classes had different demographic sensitivities, they contributed quite differently to the reduction of λ (Fig. 4). Our results showed that the first two age classes made the most important contributions to the reduction of λ in terms of the overall decrease of λ in the treated population.

## Discussion

The toxic effects of insecticides on aphid population growth rates are due to their negative effects on two individual life-cycle traits: survival probability and fertility. This study showed that these effects differed among aphid age classes; furthermore, the population growth rate, λ, was not equally sensitive to changes in the different vital rates. Several studies have shown that susceptibility to toxic effects varies among different aphid life stages (Stark and Wennergren 1995; Walthall and Stark 1997). Therefore, we were not surprised to find differences in λ sensitivities to vital rates that corresponded to specific life stages. Furthermore, one could expect that Cd and imidacloprid might act in a different manner. As noted by Laskowski (2001), these two toxicants represent two completely different classes of chemicals. Imidacloprid kills rapidly and degrades; in contrast, Cd slowly accumulates, does not degrade, and exhibits its effects in later age stages. Consistent with this, we found that the imidacloprid-induced reduction in survival made the highest contribution to the change in λ in the first age class, while Cd made the highest contribution to the change in λ in the second age class.

Surprising effects were found in mixtures of the two toxicants. At high Cd levels (200 mg/kg), λ was higher in the presence than in the absence of imidacloprid (200/0 < 200/4); and at high imidacloprid levels λ was higher in the presence than in the absence of Cd (0/40 < 200/40). This indicated that an interesting dose-dependent interaction must have occurred between the toxicants, which appeared to be antagonistic. Unfortunately, the study did not provide any further information that might point to possible physiological mechanisms behind these effects. However, it is important to stress that, for all four mixtures, the contributions of survival reduction to λ never exceeded those of single toxicants at the same concentration.

Unlike the contributions of survival reduction, contributions of fertility reduction to λ did not differ substantially between treatments. Whether the toxicant was applied alone or in a mixture, effects on the third age class contributed most to λ reduction. On the other hand, traditional elasticity analysis suggested that λ was most sensitive to reductions in vital rates in the first two age classes. This strongly differed from decomposition analysis results. Similar differences were reported by other authors (Salice and Miller 2003; Raimondo and McKenney 2006). However, these two methods addressed different questions about the sensitivity of organisms to toxicants. The different results were a direct consequence of the different mathematical formulations for elasticity and decomposition. As indicated in Eq. 3, the calculation of traditional elasticities depended solely on the projection matrix elements, *a*_*ij*_. The effect of the toxicant on the vital rates was not included, and thus, it did not influence the elasticity values. The toxic effects could be captured by performing an elasticity analysis for each treatment; thus, the toxic effect could be included by comparing the demographic sensitivities among the treated and untreated populations. Nevertheless, this elasticity analysis would not pinpoint the most toxicant-sensitive age classes. This fact was supported by our treatment-specific elasticity results for both survival and reproduction. As shown in Figs. 1 and 2, despite the different results expected from the two toxicants, the elasticity values were very similar. The highest elasticity values were found for reproduction in the second age class and survival from the first to the second age class. This was due to the fact that elasticities depend on the functional dependence of λ on all *a*_*ij*_ entries in addition to the local, functional relationships among the *a*_*ij*_ (Caswell 2000).

The low elasticity of environmentally sensitive life-cycle traits was previously discussed by Forbes et al. (2010). Based on published studies relevant to ecotoxicological testing, they showed that elasticity was negatively related to the life-cycle trait sensitivity to toxicants in all studies. This implied that natural selection favors reduced elasticities in life-cycle traits that are sensitive to environmental variations. It is difficult to disagree with that statement in general terms; however, our results indicated that eventual toxicant effects on λ, which are the outcome of the effects on particular vital rates combined with their elasticities, could differ substantially between fecundity and survival rates and among chemicals. For example, although the reductions in fertility indeed contributed most to Δλ in age classes of relatively low elasticity, this was not the case for survival rates. The effects of the chemicals on survival were similarly substantial in all age classes; therefore, the eventual toxic effects of all treatments showed that changes in survival made the largest contributions to Δλ in the first age class, which had the highest elasticity.

Elasticity analysis has been proven useful in ecotoxicological research. By applying a simple two-stage population model, Hansen et al. (1999a) manipulated life-history parameters measured in laboratory-reared animals to simulate potential effects of competition and predation on vital rates in order to explore how such factors might influence the sensitivity of population growth rate to toxicant-caused changes in individual life-history traits. Hansen et al. (1999a) showed that effectively predicting the population-level consequences of toxicant effects estimated on individual level can be improved by exploring the elasticity pattern of λ for the population over a range of ecological conditions. This work provides a good example of linking the effects of toxicants on individual organism performance to effects at the population level of organization. Nevertheless, one should be careful when interpreting elasticity analysis results or comparing them to outputs of different methods.

For the case of comparing the results of the decomposition and elasticity analyses, it is important to keep in mind that the underlying logic is different for the two approaches. Decomposition expresses the observed variation in λ as a function of the observed (co)variation in the vital rates; thus, its results are specific to the observed pattern of variation. Elasticity predicts the changes in λ that would result from any specified change in the vital rates; thus, its results are independent of variability patterns in the vital rates (Caswell 2000). The choice of method should depend on what it is being used to investigate. Elasticities have a place in life-history and conservation studies (see de Kroon et al*.* 2000); they are considered a good choice for identifying management targets or planning experiments (Laskowski 1997). However, in ecotoxicology, certain precautions must be taken. Salice and Miller (2003) stated that “decomposition analysis indicated that the vital rates that were altered when populations were exposed to Cd were not well predicted by elasticities”. Raimondo and McKenney (2006) concluded that “although elasticity analysis identifies the life stages that may be key targets for conservation efforts… the magnitude of change in population parameters is an equally important factor to consider during population-level risk assessment of toxicant exposure”. Hansen et al*.* (1999b) combined the two methods, rather than comparing them; they used elasticities to select sensitive stages, and decomposition analysis to determine whether the contributions of those stages were significant in reducing the population growth rate.

However, in the present study, the elasticity analysis missed the importance of the third age class, which turned out to contribute most to the λ reduction. Therefore, choosing the most important stage based solely on elasticity analysis would be misleading; for example, it could lead to the conclusion that running exposure tests for 2 weeks would be sufficient to estimate toxic effects. For the sake of designing short-term ecotoxicological tests, the LTRE decomposition analysis appeared to be an efficient method on its own. Unfortunately, it requires substantially more data than the elasticity analysis. In addition to the life history of a species, it requires at least some preliminary data on the responsiveness of the life stages/age classes. Nevertheless, in terms of ecological relevance and acceptable cost, this avenue may be the most meaningful, particularly for long lived, iteroparous organisms.

A decomposition analysis requires a LTRE; but ecotoxicological studies that incorporate LTREs are relatively scarce. Caswell (1996b) noted that “the full power of the LTRE approach… has not yet been applied to ecotoxicology, but there is no reason it cannot be”. Unfortunately, in the past 15 years, there has not been a break-through in the use of LTREs in ecotoxicology; altogether, less than 50 papers have been published on the subject. Stark et al*.* (2007) emphasized that the main disadvantage of this approach is that life table development is time consuming and expensive. However, it might be possible to run just one full LTRE per species for each major class of chemicals with the same mode of action; then, based on that foundation, we could design tests specific for one species to test each class of chemicals. We argue that the benefits of this approach may supersede its disadvantages.

## Conclusion

This study showed that a population-level interpretation of individual-level experimental results required information on two fronts; first, on how the toxicant affected the life stage under study, and second, on how sensitive λ was to changes in particular life stages. Our analysis combined experimental data on toxicant age-specific effects on vital rates with a sensitivity analysis of the aphid life history. A first order, fixed design-based LTRE (Caswell 2001) was used to identify age-specific impacts of the two toxicants. The analysis identified which vital rates made the largest contributions to the reduction of the population growth rate, ∆λ, under specific treatments.

The approach presented in this paper can be used to identify and select the most relevant life stages for individual-level toxicity tests. ERA tests should be designed to determine how toxicity in particular life stages contributes to population dynamics. This can be determined by assessing the sensitivity of λ to changes in particular vital rates combined with the degree of change in a vital rate due to toxic impact. Our study showed that these contributions were different for different chemicals; this suggested that the design of bioassays should be chemical-specific. For example, a test used for highly toxic, rapidly acting chemicals, like most organic pesticides (exemplified by imidacloprid in this study), should be designed differently from a test used for chemicals with low toxicity that accumulate in organisms, like metals (exemplified by Cd herein).
